# Light Extraction Analysis of AlGaInP Based Red and GaN Based Blue/Green Flip-Chip Micro-LEDs Using the Monte Carlo Ray Tracing Method

**DOI:** 10.3390/mi10120860

**Published:** 2019-12-07

**Authors:** Shuyu Lan, Hui Wan, Jie Zhao, Shengjun Zhou

**Affiliations:** 1 Center for Photonics and Semiconductors, School of Power and Mechanical Engineering, Wuhan University, Wuhan 430072, China; lanshuyu@whu.edu.cn (S.L.); zjie1994@whu.edu.cn (J.Z.); 2 The Institute of Technological Sciences, Wuhan University, Wuhan 430072, China; wanhui_hb@whu.edu.cn; 3 State Key Laboratory of Applied Optics, Changchun Institute of Optics, Fine Mechanics and Physics, Chinese Academy of Sciences, Changchun 130033, China

**Keywords:** micro-scale light emitting diode, light extraction efficiency, sapphire substrate, encapsulation

## Abstract

Micro-scale light emitting diodes (micro-LEDs) commonly employ a thin-film flip-chip (TFFC) structure whose substrate is lifted off by an excimer laser. However, flip-chip (FC) micro-LEDs with a substrate can provide a sharp rise on sidewall emission by increasing the sidewall area. Here, we investigate the influence of substrate thickness, encapsulation, surface texture, microstructures between the substrate and epilayer, as well as the size, cutting shape, and angle of the chip on the light extraction efficiencies (LEEs) of FC micro-LEDs by using the Monte Carlo ray tracing method. We find that the LEE of the blue FC micro-LED chip increases by 46.5% over that of the blue TFFC micro-LED chip. After the encapsulation with the epoxy lens is applied, the LEEs of the blue TFFC micro-LED and blue FC micro-LED increase by 129% and 110.5%, respectively. The underlying mechanisms for the use of surface texture, patterned sapphire substrate, air-void array, and chip shaping technologies to improve the LEEs of FC micro-LEDs are also investigated in detail. We find that the LEEs AlGaInP based red FC micro-LED and GaN based blue/green FC micro-LEDs exhibit a sharp rise when the chip size drops from 30 to 10 µm. The inverted trapezoid FC micro-LED with patterned sapphire substrate (PSS) and encapsulation shows extraordinarily strong top emission and high collimation. We believe that our study offers a promising and practical route for obtaining high efficiency micro-LEDs.

## 1. Introduction

Micro-scale light emitting diodes (micro-LEDs) whose size is smaller than 100 µm have been considered as the next generation display technology because of their outstanding features, such as high dynamic range, good sunlight readability, long lifetime, low power consumption, and wide color gamut [1,2,3]. Their deployment in wearable devices, cell phones, outdoor displays, and augmented reality is highly anticipated [4,5]. However, the external quantum efficiencies (EQEs) of micro-LEDs are still relatively low, which is far from satisfactory to support their potential applications. Therefore, the improvement in the EQE is an essential issue to enable micro-LEDs to have broader applications. The EQE equals the multiplication of the internal quantum efficiency (IQE) and the light extraction efficiency (LEE) [6]. Since micro-LEDs are obtained from an identical wafer as large scale LEDs, the fruitful methods for improving IQE of large scale LEDs are also applicable for micro-LEDs [7,8,9,10,11]. However, the ratio of sidewall emitting area to top emitting area is greatly different for micro-LEDs and large scale LEDs [12], so the methods applicable to large scale LEDs for improving LEE need to be reassessed in micro-LEDs. 

Recently, the light extraction efficiencies (LEEs) of LEDs have experienced a continuous progress due to the use of chip shaping, patterned sapphire substrate (PSS), air-void array (AVA), and surface texture techniques [13,14,15,16]. Furthermore, the application of encapsulation also plays an important role in enhancing LEEs of LEDs, owing to the small difference in the refractive index between sapphire substrate and encapsulation. However, a detailed study about the effect of these factors on LEEs of micro-LEDs is not available. Moreover, micro-LEDs commonly employ a thin-film flip-chip (TFFC) structure whose substrate is lifted off by an excimer laser [17,18]. However, flip-chip (FC) micro-LEDs with a substrate can provide a sharp rise on sidewall emission by increasing the sidewall area [19,20,21,22,23,24,25,26,27]. Consequently, it is of significant importance to investigate the effect of chip shaping, patterned sapphire substrate (PSS), air-void array (AVA), and surface texture on LEE of the FC micro-LED structure. Additionally, it is essential to analyze the influence of the size effect on LEEs of FC micro-LEDs when the chip size drops down to the micron scale.

In this study, we investigated the effects of substrate thickness, encapsulation, surface texture, microstructures between the substrate and epilayer, as well as the size, cutting shape, and angle of the chip on the LEEs of AlGaInP based red FC micro-LED and GaN based blue/green FC micro-LEDs (RGB FC micro-LEDs). These factors become quite critical for the LEEs of RGB FC micro-LEDs when the length and breadth of the chip are much less than its thickness. The LEE of blue FC micro-LEDs increases by 46.5% over that of the blue TFFC micro-LED. The application of chip shaping, PSS, AVA, surface texture, and encapsulation effectively improves the LEEs of RGB FC micro-LEDs. When the chip size drops from 30 to 10 µm, the LEEs of RGB FC micro-LEDs have a sharp rise. Finally, we combine all factors that contributed to the transverse-electric (TE) mode light emission and design an inverted trapezoid FC micro-LED with PSS and encapsulation. The angle of inverted trapezoid is 25°. The diameter of hemispherically shaped PSS is 2 µm, and the refractive index of encapsulation is 1.5. The inverted trapezoid FC micro-LED with PSS and encapsulation shows extraordinarily strong top emission and high collimation. Our analysis paves the way for the realization of high efficiency RGB FC micro-LEDs for potential applications in high resolution displays and augmented reality.

## 2. Simulation Model

Our simulation was performed by using Light Tools (LTs) software based on the Monte Carlo ray tracing method. Monte Carlo ray tracing is one of the most accurate ways to simulate the light propagation in LEDs [28,29,30]. Monte Carlo ray-tracing is commonly used in the case that the spontaneous photons are arbitrarily emitted from the active layer and only the geometric optics are considered in the light–structure interaction, which is usually valid when the size of the microstructure is much larger than the wavelength of light. 

**Figure 1:**
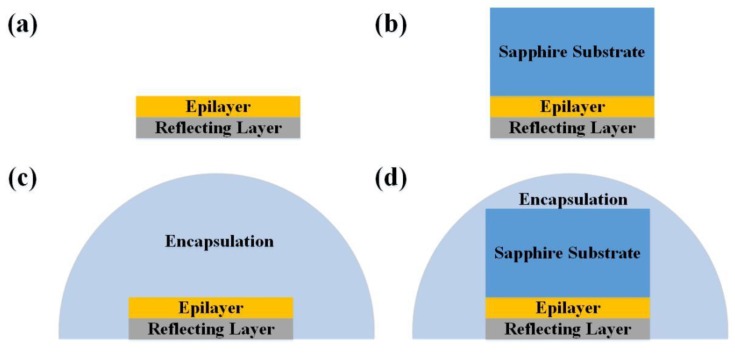
(**a**) Bare thin-film flip-chip (TFFC) micro-LED chip. (**b**) Bare FC micro-LED chip. (**c**) Encapsulated TFFC micro-LED. (**d**) Encapsulated FC micro-LED.

Figure 1 shows four types of micro-LEDs in the simulation model: TFFC micro-LEDs with and without encapsulation and FC micro-LEDs with and without encapsulation. The dimensions of micro-LEDs were 80 × 80 µm^2^. The AlGaInP based red FC micro-LED structure consisted of a metal layer, a GaInP etching stop layer, an n^+^-GaAs contact layer, n-cladding AlGaInP, an n-type AlInP diffusion barrier, GaInP/AlGaInP multiple quantum wells, a p-type AlInP diffusion barrier, p-cladding AlGaInP, and a p-GaP window layer [31,32]. The sapphire substrate was bonded onto the AlGaInP based red TFFC micro-LED to form the AlGaInP based red FC micro-LED [33]. The GaN based green/blue FC micro-LEDs structure comprised a metal layer, p-type GaN, a p-AlGaN electron blocking layer, InGaN/GaN multiple quantum wells, n-type GaN, and sapphire substrate [34,35,36,37,38].

The optical parameters of the layers of RGB micro-LEDs are shown in Table 1 [1]. The central wavelengths were 622 µm, 527 µm, and 470 µm for RGB micro-LEDs. The encapsulation with the epoxy lens was designed as a hemisphere. To ensure that most of the photons could escape into the air without multiple reflections in the encapsulation, the refractive index and diameter of the epoxy lens were set to 1.5 and 1 cm [6], respectively. The bottoms of FC micro-LEDs were equipped with a reflecting layer.

## 3. Results and Discussion

**Figure 2:**
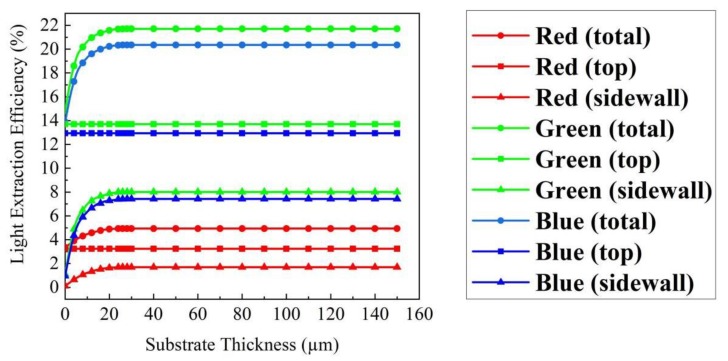
LEEs of each face of RGB FC micro-LEDs with variable thicknesses of sapphire substrate.

Figure 2 shows the relationship between substrate thickness and the LEEs of each face of RGB micro-LEDs. The sidewall and total LEEs of RGB FC micro-LEDs remained constant when the substrate thickness was larger than 28 µm. When the substrate thickness changed from 0 to 28 µm, the sidewall and total LEEs increased with the substrate thickness. However, the top LEEs of RGB FC micro-LEDs were almost constant when the substrate thickness changed from 0 to 150 µm, indicating that the variation of total LEEs depended on the change of sidewall LEEs. Owing to the high extinction coefficient (k) of AlGaInP materials, the LEEs of AlGaInP based red FC micro-LEDs were markedly lower than those of GaN based green/blue FC micro-LEDs. In addition, the LEE of the blue FC micro-LED increased by 46.5% over that of the blue TFFC micro-LED.

**Figure 3:**
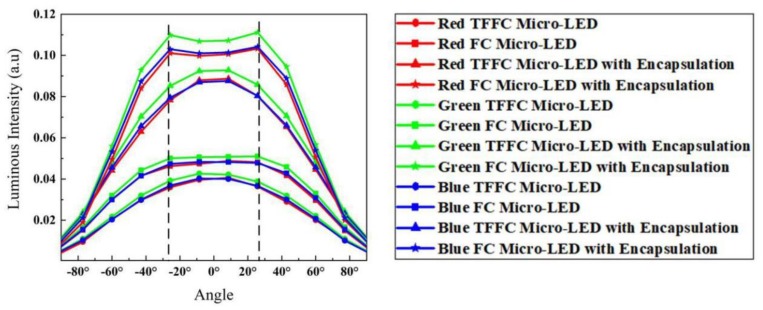
Luminous intensities of four types of RGB micro-LEDs: TFFC micro-LEDs, FC micro-LEDs, TFFC micro-LEDs with encapsulation, and FC micro-LEDs with encapsulation.

Figure 3 shows the luminous intensities of four types of RGB micro-LEDs. The luminous intensities of encapsulated RGB micro-LEDs with the epoxy lens were almost double that of bare RGB micro-LED chips. Actually, when the encapsulation was applied, the LEEs of the blue TFFC micro-LED and blue FC micro-LED increased by 129% and 110.5%, respectively. Additionally, the sidewall emissions of RGB FC micro-LEDs exhibited a significant enhancement due to the thick, transparent sapphire substrate and epoxy lens, leading to the highest peak luminous intensities of encapsulated RGB FC micro-LEDs at ± 27°.

**Figure 4:**
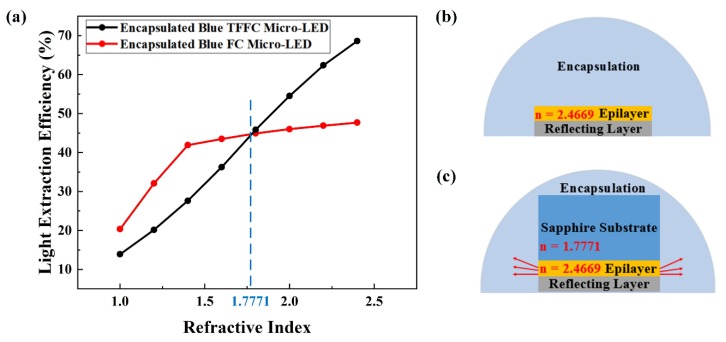
(**a**) LEEs of the encapsulated blue TFFC micro-LED and encapsulated blue FC micro-LED with variable refractive indices of encapsulation. (**b**) Encapsulated blue TFFC micro-LED and (**c**) encapsulated blue FC micro-LED.

Figure 4a shows the relationship between the LEEs of encapsulated blue micro-LEDs and the refractive index of encapsulation. Actually, a smaller refractive index difference led to a larger escape cone, and more photons could escape into the air. Therefore, as shown in Figure 4a, the LEE of the encapsulated blue TFFC micro-LED in Figure 4b kept increasing when the refractive index of encapsulation changed from one to 2.4. However, for the encapsulated blue FC micro-LED, we could see a significant change on the rise pace at the refractive index of 1.4. The angle of total internal reflection (TIR) at the sapphire substrate–encapsulation interface was 52° when the refractive index of encapsulation was 1.4, which ensured that all photons in the sapphire substrate escaped into the encapsulation. Thereby, when the refractive index of encapsulation was smaller than 1.4, photons could escape from the top and sidewall of sapphire substrate, as well as the sidewall of the epilayer. When the refractive index of encapsulation was larger than 1.4, the remaining photons were in the epilayer of the micro-LED, and only the sidewall emission of the epilayer had a slow rise, as shown by the red lines in Figure 4c. Due to the thin epilayer and loss of top emission, the LEE of micro-LED exhibited a slow rise pace when the refractive index was larger than 1.4. Moreover, the encapsulated blue TFFC micro-LED and encapsulated blue FC micro-LED had the same LEE when the refractive index of encapsulation was 1.7771, since they were the same structure at this point. However, when the refractive index of encapsulation was larger than 1.7771, sapphire substrate would limit the rise of LEE because of the TIR at the epilayer–substrate interface.

**Figure 5:**
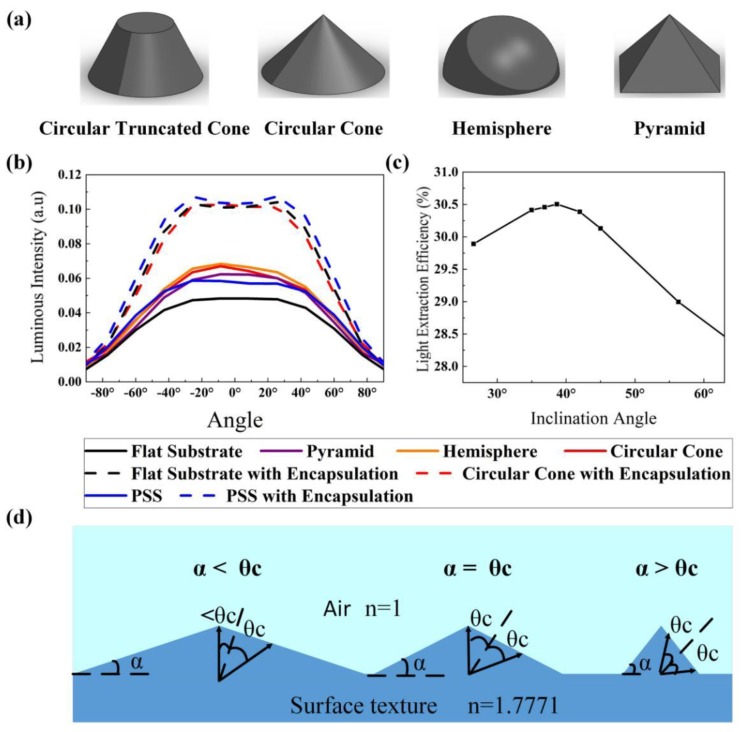
(**a**) Shapes of the microstructure including a circular truncated cone, circular cone, hemisphere, and pyramid. (**b**) Luminous intensities of bare and encapsulated blue FC micro-LEDs with different microstructures. (**c**) LEEs of blue FC micro-LEDs with variable slanted angles of the circular cone. (**d**) Analysis of the effect of the slanted angles of the circular cone on the LEEs of blue FC micro-LEDs. α is the slanted angles of the circular cone, and θc is the angle of total internal reflection (TIR).

In order to further enhance the LEEs of FC micro-LEDs, we introduced a special microstructure on the top surface of sapphire substrate, which was the so-called surface texture. Many photons scattered at the texture interface and emitted into the air, resulting in an enhancement of the LEEs of FC micro-LEDs. We explored the effect of the shapes of microstructures, including a circular truncated cone, circular cone, hemisphere, and pyramid, as shown in Figure 5a, on the LEEs of FC micro-LEDs. The heights and base areas of these microstructures were set to 1 µm and 3.14 µm^2^, respectively. As shown in Figure 5b, the luminous intensities of blue FC micro-LEDs with textured substrate were higher than that of the blue FC micro-LED with flat substrate. Since the curved sidewall can contribute to the escape of photons, the luminous intensity of the blue FC micro-LED with a circular truncated cone, circular cone, or hemisphere as the shape of the surface texture was slightly higher than that of the blue FC micro-LED with a pyramid as the shape of the surface texture. Moreover, a circular cone was chosen to be the shape of surface texture to explore the effect of the slanted angle on the LEE of the blue FC micro-LED. In Figure 5c, we find that the slanted angle of 38° was the best angle for enhancing the LEEs of blue FC micro-LEDs.

As shown in Figure 5d, when the slanted angles of the circular cone (α) were smaller than the angle of TIR (θc = 34°), the range of incident angles of photons that could escape into the air was less than 2θc. When α increased from θc to 90° − θc, the LEEs would be constant since the ranges of the incident angles of photons that could escape into the air were the same (2θc) [39]. However, due to the sidewall emission of photons at a large incident angle, the photons at a small incident angle were more than those at a large incident angle at the layer of the surface texture. Thereby, the maximum LEE was only obtained at the slanted angle of θc. Taking other factors into consideration, the simulation result of 38° was reasonable. Moreover, we investigated the effect of the surface texture on the LEEs of encapsulated blue FC micro-LEDs. As shown by the dashed lines in Figure 5b, the effect of surface texture on the LEEs of encapsulated blue FC micro-LEDs was almost eliminated, since the refractive index difference between sapphire substrate and encapsulation was only 0.2. Most of the photons could escape from the substrate into the encapsulation even without the surface texture. Furthermore, we analyzed the effect of PSS on the LEEs of FC micro-LEDs. As shown in Figure 5b, the PSS could effectively increase the luminous intensity of the blue FC micro-LED with or without encapsulation, revealing that PSS was more efficient than the surface texture in an encapsulated LED. 

**Figure 6:**
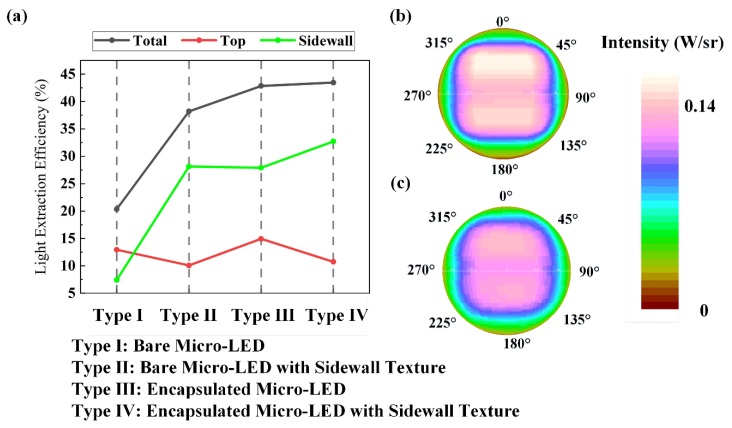
(**a**) LEEs of each face (total, top, sidewall) of blue micro-LEDs. (**b**) Top luminous intensity distribution of the encapsulated blue FC micro-LED. (**c**) Top luminous intensity distribution of the encapsulated blue FC micro-LED with the sidewall texture.

Figure 6a shows the effect of sidewall texture on the LEEs of each face of the blue FC micro-LED. The sidewall texture significantly increased the sidewall LEE and total LEE of the bare FC micro-LED. When the encapsulation was applied, the total LEE of the FC micro-LED with the sidewall texture was the same as that of the FC micro-LED without the sidewall texture, whereas the sidewall LEE of the FC micro-LED with the sidewall texture was higher than that of the FC micro-LED without the sidewall texture. In Figure 6b,c, the top luminous intensity of the encapsulated FC micro-LED without the sidewall texture is much higher than that of the encapsulated FC micro-LED with the sidewall texture. Therefore, in the case of encapsulation, the sidewall texture was more suitable for transverse-magnetic (TM) mode light emission rather than transverse-electric (TE) mode light emission due to the great enhancement in sidewall emission rather than top emission.

**Figure 7:**
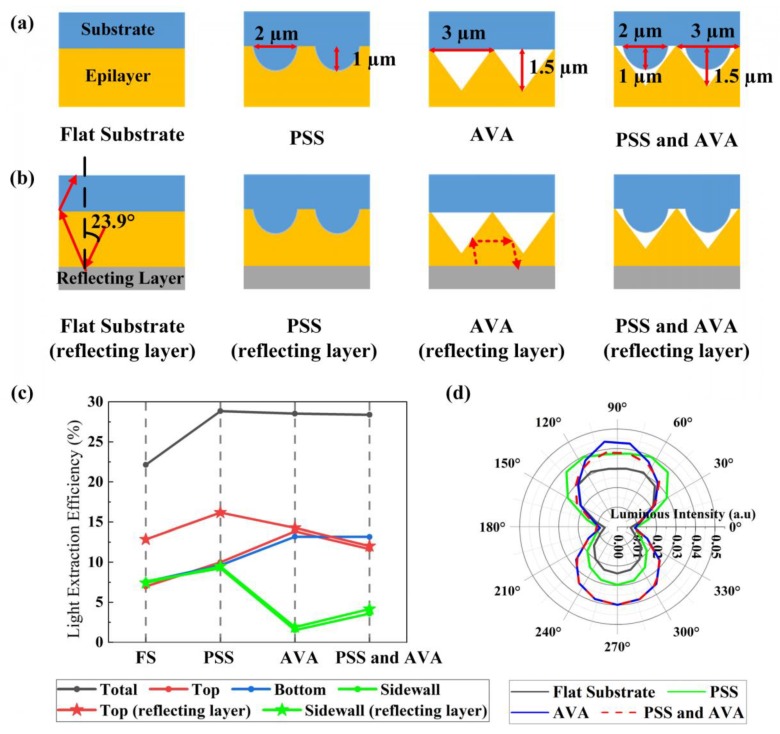
(**a**) Blue micro-LEDs with the flat substrate, PSS, air-void array (AVA), or both (PSS and AVA), but without the reflecting layer. (**b**) Blue micro-LEDs with the flat substrate, PSS, AVA, or both (PSS and AVA) and with the reflecting layer. (**c**) LEEs of each face (total, top, bottom, sidewall) of blue micro-LEDs. (**d**) Light fields of blue micro-LEDs without the reflecting layer.

Figure 7a shows the effect of PSS and AVA on the LEEs of each face of blue FC micro-LEDs. Four types of blue micro-LEDs structures were considered: a micro-LED with a flat substrate, a micro-LED with PSS, a micro-LED with AVA, and a micro-LED with PSS and AVA. The diameter of the hemispherically shaped sapphire patterns was set to 2 µm. The air-voids were set as triangular prisms with a cross-section of 3 µm in length and 1.5 µm in height. A reflecting layer was deposited onto the GaN epilayer, as shown in Figure 7b. Figure 7c shows the LEEs of each face of the blue micro-LEDs in Figure 7a,b. For the micro-LEDs without the reflecting layer, the LEEs of each face of the micro-LED with the flat substrate or with PSS were almost the same. The LEEs of each face of the micro-LED with PSS were much higher than those of micro-LED with the flat substrate, indicating that PSS improved the LEEs of each face simultaneously. The micro-LED with AVA had significantly high top and bottom LEEs, but an extremely low sidewall LEE, indicating that AVA enhanced the top and bottom LEEs, but reduced the sidewall LEE, which led to a higher collimated light emission. Moreover, the top and bottom LEEs of the micro-LED with both PSS and AVA were larger than those of the micro-LED with PSS, while the sidewall LEE of the micro-LED with both PSS and AVA was larger than that of the micro-LED with AVA. As a result, the light field of the micro-LED with both PSS and AVA exhibited a uniform distribution, as in Figure 7d, which could lead to a small angular color shift [2]. The micro-LED with AVA had a strong enhancement in top and bottom luminous intensities, while the micro-LED with PSS improved the luminous intensity of each direction simultaneously.

Compared to micro-LEDs without a reflecting layer, the sidewall LEEs of micro-LEDs with the reflecting layer were almost constant. Since the TIR angle of the epilayer–air interface was 23.9° (Figure 7b), the photons with an incident angle larger than 23.9° at the bottom epilayer–air interface were reflected due to the TIR, which had no effect on the variation of LEEs when a reflecting layer was added. The photons with an incident angle from 0° to 23.9° were reflected by the reflecting layer, and their incident angles at the sidewall and top interface were in the range of 66.1°–90° and 0°–23.9°, respectively. Therefore, they could not emit from the sidewall surface, but could escape from the top surface. However, as shown by the red dashed lines in Figure 7b, the photons reflected by the reflecting layer had an incident angle between 21.1° and 45° at the slanted planes of AVA. As a result, most of the photons reflected by the reflecting layer could not escape from the top surface. Consequently, the sidewall and top LEEs of the micro-LED with AVA or the micro-LED with PSS and AVA were almost constant when a reflecting layer was added.

**Figure 8:**
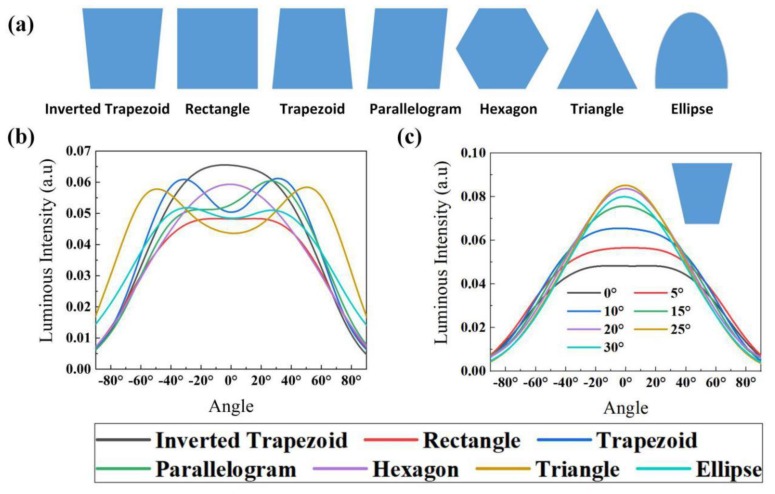
(**a**) Cross-sectional images of chip cutting shapes. Luminous intensities of blue FC micro-LEDs: (**b**) with different cutting shapes of the chip; (**c**) with different cutting angles of the inverted trapezoid-chip.

The cutting shape and angle of the chip also played an important role in improving the LEEs of FC micro-LEDs, since they could change the track of photon propagation. We simulated different cutting shapes of the chip to explore the effect of cutting shapes on the LEEs of FC micro-LEDs. Figure 8a shows the cross-sectional images of chip cutting shapes, including inverted trapezoid, rectangle, trapezoid, parallelogram, hexagon, triangle, and ellipse. In Figure 8b, the luminous intensity of a rectangular blue FC micro-LED was the lowest in these cases. The parallelogram-chip led to an asymmetric light field. Moreover, the light field of the blue FC micro-LED with a cutting shape of a rectangle, hexagon, or inverted trapezoid exhibited a Lambertian distribution, which was suitable for TE mode light emission, while the cutting shape of a trapezoid, triangle, or ellipse substrate was suitable for TM mode light emission due to the strong sidewall emission. We changed the cutting angle of the inverted trapezoid-chip to explore the effect of chip cutting angles on the LEEs of FC micro-LEDs. As shown in Figure 8c, the luminous intensity patterns of micro-LEDs became more collimated when the cutting angle changed from 0° to 20°, which meant more photons emitted to the air at a smaller angle. However, the luminous intensity reduced when the cutting angle was larger than 25°. Hence, it was better to choose a cutting angle in the range of 20° to 25°.

**Figure 9:**
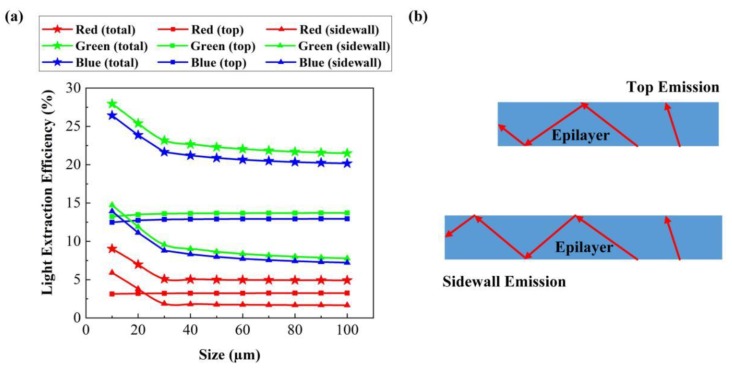
(**a**) LEEs of each face of RGB FC micro-LEDs with variable chip size. (**b**) Propagation pathways of photons in FC micro-LEDs with different chip sizes.

Figure 9a shows the effect of the chip size on the LEE of RGB FC micro-LEDs. we kept the height of RGB FC micro-LEDs at 126 µm and changed the chip size from 10 to 100 µm. When the chip size ranged from 10 to 100 µm, the sidewall and total LEEs of RGB FC micro-LEDs decreased, while the top LEEs were almost constant, indicating that the variation of total LEEs depended on the change of sidewall LEEs. Figure 9b shows the propagation pathways of photons in FC micro-LEDs with different chip sizes. As shown in Figure 9b, the light propagation pathway of sidewall emission increased with the chip size, resulting in more photons being absorbed by the epilayer of RGB FC micro-LEDs. However, the increase of the chip size had no effect on the top emission. Additionally, when the chip size dropped from 30 to 10 µm, the sidewall and total LEEs of RGB FC micro-LEDs had a sharp rise. For a deep ultraviolet LED with TM polarized light emission, this phenomenon would be more obvious.

**Figure 10:**
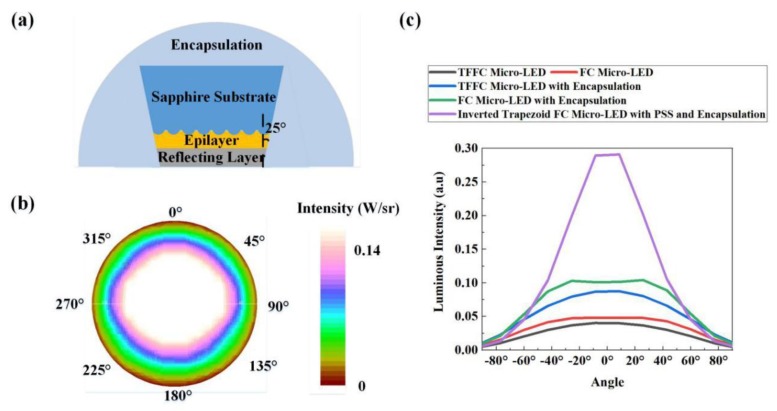
(**a**) Inverted trapezoid FC micro-LED with PSS and encapsulation. (**b**) Top luminous intensity distribution of the inverted trapezoid FC micro-LED with PSS and encapsulation. (**c**) Comparison between the luminous intensities of the inverted trapezoid FC micro-LED with PSS and encapsulation, TFFC micro-LED, FC micro-LED, TFFC micro-LED with encapsulation, and FC micro-LED with encapsulation.

We combined all factors that contributed to the TE mode light emission and designed an inverted trapezoid FC micro-LED with PSS and encapsulation as shown in Figure 10a.The angle of the inverted trapezoid was 25°. The diameter of the hemispherically shaped PSS was 2 µm, and the refractive index of encapsulation was 1.5. Figure 10b shows the significantly high top luminous intensity of the optimized FC micro-LED. In Figure 10c, the inverted trapezoid FC micro-LED with PSS and encapsulation exhibited much stronger top emission and higher collimation than the TFFC micro-LED with or without encapsulation and the FC micro-LED with or without encapsulation. This result revealed that the inverted trapezoid FC micro-LED with PSS and encapsulation was extremely suitable for applications in high resolution displays and augmented reality. Actually, compared to these four types of micro-LEDs, the LEE of the inverted trapezoid FC micro-LED with PSS and encapsulation increased by 231.9%, 126.5%, 44.9%, and 7.6%.

## 4. Conclusions

In summary, we investigated the influence of substrate thickness, encapsulation, surface texture, microstructures between the substrate and epilayer, as well as the size, cutting shape, and angle of the chip on the LEEs of FC micro-LEDs by using the Monte Carlo ray tracing method. For GaN based blue micro-LEDs, the LEE of the bare FC micro-LED chip increased by 46.5% over that of the bare TFFC micro-LED chip. After the encapsulation with the epoxy lens was applied, the LEEs of the blue TFFC micro-LED and blue FC micro-LED increased by 129% and 110.5%, respectively. However, the effect of the surface texture on the LEEs of encapsulated micro-LEDs was almost eliminated due to the small refractive index difference between sapphire substrate and encapsulation. The PSS technology could increase the LEE when encapsulation was applied, revealing that PSS was more efficient than the surface texture in an encapsulated LED. The PSS technology improved the top, bottom, and sidewall LEEs of the micro-LED simultaneously, and the AVA had a strong enhancement of the top and bottom LEEs, but a decrease of the sidewall LEE, thereby leading to a higher collimated light emission. Moreover, the combination of PSS and AVA brought a uniform distribution on the light field whose angular color shift was small. Additionally, an inverted trapezoid chip with an angle within 20° to 25° brought a larger luminous intensity and smaller emit angle. For the AlGaInP based red FC micro-LED and GaN based blue/green FC micro-LEDs, the LEEs had a sharp rise when the chip size dropped from 30 to 10 µm. The present findings emphasized the importance of FC structure for improving the LEEs of micro-LEDs, which paves the way for high efficiency micro-LEDs. 

## Figures and Tables

**Table 1:** Optical parameters of the materials in the simulation model.

Materials	Thickness (µm)	Central Wavelengths (µm)	Refractive Index n	Extinction Coefficient k
Epilayer of red micro-LED	6	622	3.3315	0.02
Sapphire substrate of red micro-LED	120	622	1.7664	
Epilayer of green micro-LED	6	527	2.4263	0.003
Sapphire substrate of green micro-LED	120	527	1.7721	
Epilayer of blue micro-LED	6	470	2.4669	0.003
Sapphire substrate of blue micro-LED	120	470	1.7771	
